# Role of CD133/NRF2 Axis in the Development of Colon Cancer Stem Cell-Like Properties

**DOI:** 10.3389/fonc.2021.808300

**Published:** 2022-01-26

**Authors:** Jimin Park, Seung Ki Kim, Steffanus Pranoto Hallis, Bo-Hyun Choi, Mi-Kyoung Kwak

**Affiliations:** ^1^ Department of Pharmacy and BK21FOUR Advanced Program for SmartPharma Leaders, Graduate School of The Catholic University of Korea, Gyeonggi-do, South Korea; ^2^ Department of Pharmacology, School of Medicine, Daegu Catholic University, Daegu, South Korea; ^3^ Integrated Research Institute for Pharmaceutical Sciences, The Catholic University of Korea, Gyeonggi-do, South Korea; ^4^ College of Pharmacy, The Catholic University of Korea, Gyeonggi-do, South Korea

**Keywords:** CD133, cancer stem cell, NRF2, PI3K/AKT/GSK-3β, sphere formation, colorectal cancer

## Abstract

Cancer stem cells (CSCs) exhibit intrinsic therapy/stress resistance, which often cause cancer recurrence after therapy. In this study, we investigated the potential relationship between the cluster of differentiation (CD)-133, a CSC marker of colon cancer, and nuclear factor erythroid 2-like 2 (NFE2L2; NRF2), a master transcription factor for the regulation of multiple antioxidant genes. In the first model of CSC, a sphere culture of the colorectal cell line HCT116, showed increased levels of CD133 and NRF2. Silencing of *CD133* reduced the levels of CSC markers, such as Kruppel-like factor 4 (KLF4) and ATP-binding cassette subfamily G member 2 (ABCG2), and further suppressed the expression levels of NRF2 and its target genes. As a potential molecular link, CD133-mediated activation of phosphoinositide 3-kinase/serine-threonine kinase (PI3K/AKT) signaling appears to increase the NRF2 protein levels *via* phosphorylation and the consequent inhibition of glycogen synthase kinase (GSK)-3β. Additionally, *NRF2*-silenced HCT116 cells showed attenuated sphere formation capacity and reduced CSC markers expression, indicating the critical role of the NRF2 pathway in the development of CSC-like properties. As a second model of CSC, the CD133^high^ cell population was isolated from HCT116 cells. CSC-like properties, including sphere formation, motility, migration, colony formation, and anticancer resistance, were enhanced in the CD133^high^ population compared to CD133^low^ HCT116 cells. Levels of NRF2, which were elevated in CD133^high^ HCT116, were suppressed by *CD133*-silencing. In line with these, the analysis of The Cancer Genome Atlas (TCGA) database showed that high levels of CD133 expression are correlated with increased NRF2 signaling, and alterations in *CD133* gene or expression are associated with unfavorable clinical outcome in colorectal carcinoma patients. These results indicate that the CD133/NRF2 axis contributes to the development of CSC-like properties in colon cancer cells, and that PI3K/AKT signaling activation is involved in CD133-mediated NRF2 activation.

## Introduction

Cancer stem cell (CSC) is a subpopulation of tumor cells, which is known to account for 1–2% of tumors. Initially, Dick and colleagues identified the leukemia-initiating cluster of differentiation (CD)-34^+^ CD38^-^ cells from acute myeloid leukemia (AML) and showed that these cell fractions have differentiation and self-renewal capacities using serial transplantation in immunodeficient mice (1). CSCs share several common characteristics with normal stem cells, including self-renewal capacity, asymmetric division, and differentiation potential (2–4). In addition, CSCs attribute core characteristics to aggressive cancers due to their intrinsic resistance to anticancer treatment. Upregulation of drug efflux transporters, increased expression of the reactive oxygen species (ROS) scavenging system, and promotion of DNA damage repair are observed in CSCs, which enhance their survival in response to chemo- and radiotherapy (5–9). Several cell surface molecules, such as CD44, CD133, and ATP-binding cassette subfamily G member 2 (ABCG2), and transcription factors, such as Kruppel-like factor 4 (KLF4) and octamer-binding transcription factor 4 (OCT4), have been used to isolate and characterize CSCs (2, 8, 10, 11)

CD133 (Prominin-1) is a transmembrane penta-span glycoprotein localized in cholesterol-based lipid rafts in the plasma membrane (12). It has two large extracellular loops, an N-terminal extracellular domain and a C-terminal intracellular domain, and eight glycosylation sites. Since its first identification in human hematopoietic stem cells (13), CD133 has been recognized as a marker of CSCs (14). The CD133-positive population was identified as 2.5% of the total tumor cells from colon cancer tissues and reproduced original tumors in immunodeficient mice (15). In an animal model of renal capsule transplantation, all colon cancer-initiating cells were CD133-positive, while CD133-negative cells, which comprised majority of cancer specimens, were not able to initiate tumorigenesis (16). High CD133 expression in colorectal cancer is correlated with low survival of patients with cancer (17). Furthermore, CD133 expression is associated with an aggressive cancer phenotype. CD133 overexpression in pancreatic cancer cells induced epithelial-mesenchymal transition (EMT) and enhanced cancer metastasis in athymic mice (18). CD133-positive cells from primary non-small cell lung cancer (NSCLC) specimens exhibited higher levels of genes associated with stemness, migration, and drug efflux than CD133-negative cells (19). Additionally, cisplatin treatment in primary tumor xenografts showed that the CD133-positive population survived after therapy. Suppression of ABCG2 in CD133-positive colon cancer cells enhances their apoptotic response to chemotherapy (20).

Nuclear factor erythroid 2-like 2 (NFE2L2/NRF2) is a cap’n’collar (CNC) transcription factor containing a basic leucine zipper (bZip) domain. Under normal conditions, NRF2 binds to the cytoplasmic protein, Kelch-like ECH-associated protein (KEAP1) and is subjected to proteasomal degradation *via* the formation of the KEAP1/Cul3/Rbx1 E3 ligase complex (21). In the presence of oxidative/electrophilic stress, NRF2 is liberated from KEAP1 and translocated into the nucleus where it binds to the antioxidant response element (ARE) of the promoter regions of an array of genes (22, 23). These genes encode various cytoprotective proteins, including detoxifying enzymes (e.g., NAD(P)H: quinone oxidoreductase-1 [NQO1], aldo-keto reductase 1C1 [AKR1C1]), antioxidant proteins (*e.g.*, glutamate-cysteine ligase catalytic subunit [GCLC], glutathione peroxidase 1 [GPX1]), heme metabolizing enzymes (*e.g.*, heme oxygenase-1 [HO-1]), and drug efflux transporters (*e.g.*, breast cancer resistance protein [BCRP/ABCG2], multi-drug resistance-1 [MDR1/ABCB1]) (21, 23, 24). NRF2 is accepted as a critical component of cellular defense systems that cope with oxidative and environmental stress by removing intracellular ROS/electrophiles, thereby maintaining cellular redox homeostasis (25, 26). In addition to KEAP1, glycogen synthase kinase-3β (GSK-3β), a Ser/Thr kinase, also participates in NRF2 regulation (27, 28). GSK-3β phosphorylates NRF2 and promotes β-transducin repeat-containing protein (β-TRCP)-dependent ubiquitination and subsequent proteasomal degradation (27, 29). Since GSK-3β activity is inhibited by phosphoinositide 3-kinase (PI3K)/protein kinase B (AKT)-mediated phosphorylation, PI3K/AKT activation results in the blockade of β-TRCP-dependent NRF2 degradation (27, 30).

Although NRF2 shows protective roles in normal cells under stressful conditions, elevated levels of NRF2 in cancers promote cancer cell survival and facilitate tumor growth, cancer progression, and development of resistance to therapy (31–33). In particular, there is evidence that NRF2 signaling is upregulated in several types of CSC models, such as tumor spheres, CD44^high^ cells, and aldehyde dehydrogenase (ALDH)^high^ cancer cells. Additionally, this upregulation was responsible for the development of CSC-like properties, including therapy resistance, spheroid growth, enhanced migration capacity, and facilitated tumor growth (34–39). In the present study, we investigated the potential relationship between CSC markers, CD133 and NRF2, in colon cancer cells and demonstrated the role of the CD133/NRF2 axis in the development of CSC-like properties using two CSC models of spheroid culture system and CD133^high^ subpopulation system.

## Materials and Methods

### Materials

Doxorubicin, 3-(4,5-dimethylthiazol-2-yl)-2,5-diphenyltetrazolium bromide (MTT), and LY294002 were purchased from Sigma-Aldrich (St. Louis, MO, USA). Lipofectamine RNA iMAX was purchased from Invitrogen Life Technologies (Carlsbad, CA, USA). Agarose was purchased from Promega Corp. (Madison, WI, USA). Antibodies recognizing NRF2 (sc-13032), NQO1 (sc-16464), and glyceraldehyde 3-phosphate dehydrogenase (GAPDH; sc-47724) were obtained from Santa Cruz Biotechnology (Dallas, TX, USA). Antibodies against AKT (#4691S), p-AKT (S473; #4060S), GSK-3β (#9315S), p-GSK-3β (S9; #9336S), KLF4 (#4038S), ABCG2 (#4477S), and CD133 (#5860S) were purchased from Cell Signaling Technology (Danvers, MA, USA). Anti-AKR1C1 (H00001645-B01P), anti-GCLC (ab207777), and anti-HO-1 (ADI-SPA-896) antibodies were purchased from Abnova (Walnut, CA, USA), Abcam (Cambridge, UK), and Enzo Life Sciences (Farmingdale, NY, USA), respectively. Allophycocyanin (APC)-conjugated CD133 (25-110-963-110-963) antibody and its control IgG (25-113-434-113-434) were purchased from Miltenyi Biotec (Bergisch Gladbach, NW, Germany). TB Green Premix Ex Taq was obtained from Takara Bio (Kusatsu, Shiga, Japan). Ultra-low attachment culture dishes or 6-well plates for sphere culture were obtained from Corning Costar Corp. (Cambridge, MA, USA). Gene-specific small interfering RNAs (siRNAs), CD133 siRNA, NRF2 siRNA, and non-specific scrambled control siRNA, were obtained from Bioneer Corp. (Daejeon, Republic of Korea).

### Cell Culture

The human colorectal carcinoma cell line, HCT116, was purchased from the American Type Culture Collection (Rockville, MD, USA). Cells were cultured in Dulbecco’s modified Eagle’s medium (DMEM; Welgene Inc., Daegu, Republic of Korea) and Nutrient Mixture F-12 medium (Welgene Inc.) supplemented with 10% fetal bovine serum (FBS; Corning Costar Corp.) and 1% penicillin/streptomycin (Welgene Inc.). The cells were grown at 37°C in a humidified atmosphere containing 5% carbon dioxide (CO_2_). Colorectal cancer cell line Colo205, pancreatic carcinoma cell line PANC-1 and MIA PaCa-2, and breast carcinoma cell line MCF-7 were purchased from ATCC, and were grown in RPMI1640 (Colo205) and DMEM (PAC-1, MIA PaCa-2, MCF-7).

### Sphere Culture

Cells were plated at a density of 1 × 10^5^ cells/mL in ultralow attachment 100 mm plates or 6-well plates. The cells were grown in serum-free DMEM and Nutrient Mixture F-12 medium supplemented with B27 (Life Technologies), 20 ng/mL epithelial growth factor, 20 ng/mL basic fibroblast growth factor (R&D Systems, Minneapolis, MN, USA), 5 mg/mL bovine insulin (Cell Applications Inc., San Diego, CA, USA), 0.5 mg/mL hydrocortisone (Sigma-Aldrich), and penicillin/streptomycin. In sphere culture conditions, HCT116 cells were grown for 3–6 d and then harvested as described previously (37). The sphere number and size were counted using ToupView software (ToupTek, Hangzhou, Zhejiang, China).

### siRNA Transfection

Cells were seeded at a density of 2×10^5^ cells in a 60 mm dish and grown for 2 d. The medium was changed to antibiotic-free DMEM and Nutrient Mixture F-12 medium with 10% FBS. The cells were transfected with final concentration of 10 nmol predesigned *CD133*-specific siRNA (3′-GUCUACAAGGACUUUCCAA-5′ and 3′-UUGGAAAGUCCUUGUAGAC-5′) or *NRF2*-specific siRNA (3′-GAGACUACCAUGGUUCCAA-5′ and 3′-UUGGAACCAUGGUAGUCUC-5′), or scrambled control siRNA using (1:3) volume ratio of Lipofectamine RNAiMAX reagent (Invitrogen Life Technologies) according to manufacturer’s protocol. After 24 h, the transfection complex-containing medium was removed, and the cells were further cultured for 24 h for recovery in complete medium (36).

### Immunoblotting Analysis

Whole lysates were prepared by adding 5X sample buffer containing 250 mM Tris-HCl (pH 6.8), 10% sodium dodecyl sulfate (SDS; Biosesang, Gyeonggi-do, Korea), 30% glycerol, 0.25% bromophenol blue, and 5% β-mercaptoethanol (Sigma-Aldrich, Co.). Protein samples were separated in 8–10% SDS-polyacrylamide gels and then transferred to nitrocellulose membranes (Whatman GmbH, Dassel, Germany). The membranes were blocked with 5% skim milk for 1 h and then incubated with the primary antibody in 3% bovine serum albumin (BSA) overnight. After incubation with the secondary antibody, chemiluminescent images were detected using a LAS-4000 mini-imager (GE Healthcare Life Sciences, Piscataway, NJ, USA). The loading control was detected after antibodies removal using stripping buffer (Restore Western blot stripping buffer; Thermo Fischer Scientific Inc., Waltham, MA, USA) followed by membrane blocking, primary and secondary antibodies incubation, and chemiluminescent detection as described previously.

### Total RNA Extraction and Real-Time Reverse Transcription-Polymerase Chain Reaction (RT-PCR) Analysis

Total RNA was isolated using TRIzol reagent (Thermo Fisher Scientific Inc., Waltham, MA, USA) and processed for cDNA synthesis. RT reactions were performed by incubating 200 ng of total RNA with a reaction mixture containing oligo (dT), Go script 5X buffer, MgCl_2_ (25 mM), and dNTP (2 mM) (Promega Corp.). Relative quantification of real-time RT-PCR was carried out using a Roche Light Cycler (Mannheim, Germany) with the Takara TB Premix ExTaq System (Otsu, Japan) as described previously (40). Primers were synthesized by Bioneer Corp., and the primer sequences for human genes were as follows: Hypoxanthine phosphoribosyltransferase-1 (*HPRT1*), 5′-TGGCGTCGTGATTAGTGATG-3′ and 5′-GCTACAATGTGATGGCCTCC-3′, 5′-CCTGGCGTCGTGATTAGTGA-3′ and 5′-GCTACAATGTGATGGCCTCC-3′, 5′-TGACACTGGCAAAACAATGC-3′ and 5′-CAAATCCAACAAAGTCTGGC-3′; *ABCG2*, 5′-CACAACCATTGCATCTTGGCTG-3′ and 5′-TGAGAGATCGATGCCCTGCTTT-3′; *KLF4*, 5′-ACACTTGTGATTACGCGGGCTGC-3′ and 5′-GGCGAATTTCCATCCACAGCCG-3′; *CD133*, 5′-CCGCAGGAGTGAATCTTTTA-3′ and 5′-CTATAGGAAGGACTCGTTGC-3′; *NRF2*, 5′-TAGCAATGAAGACTGGGCTC-3’ and 5′-CCAGTGGATCTGCCAACTAC-3′; *NQO1*, 5′-CAGTGGTTTGGAGTCCCTGCC-3’ and 5′-TCCCCGTGGATCCCTTGCAG-3′; *AKR1C1*, 5′-GAAAGAAACATTTGCCAGCC-3’ and 5′-TGAGCAGAATCAATATGGCG-3′; *GCLC*, 5′-TGAAGGGACACCAGGACAGCC-3’ and 5′-GCAGTGTGAACCCAGGACAGC-3′; *HO-1*, 5′-GCTGCTGACCCATGACACCAAGG-3′ and 5′-AAGGACCCATCGGAGAAGCGGAG-3′; *GPx1*, 5′-TTCCCGTGCAACCAGTTTG-3′ and 5′-TTCACCTCGCACTTCTCGAA-3′.

### Flow Cytometry and Cell Sorting

Approximately 1×10^6^ HCT116 cells were harvested and incubated in 2 μL CD133/1-APC staining dye (Miltenyi Biotec) with 98 μL buffer containing 2 mM ethylenediaminetetraacetic acid (EDTA) and 2% FBS for 40 min. IgG control samples were incubated with 2 μL REA control (S)-APC staining dye (Miltenyibiotec Korea) with 98 μL of buffer. The fluorescence intensity of the stained cells was analyzed using an FACS Aria III cell sorter flow cytometer (BD Biosciences, Franklin Lakes, NJ, USA), and CD133^high^ and CD133^low^ subpopulations were sorted as described previously (35).

### MTT Assay

Cells were plated at a density of 3×10^3^ cells/well in a 96-well plate and incubated with doxorubicin for 24 h. After the addition of MTT solution (2 mg/mL), the cells were further incubated for 4 h. The MTT solution was removed, 100 μL/well of dimethyl sulfoxide (DMSO; Sigma-Aldrich Co.) was added, and the absorbance was measured at 540 nm using SpectraMax (Molecular Devices, San Jose, CA, USA) (41).

### Wound Healing Assay

To determine cell motility, CD133^high^ and CD133^low^ cells were plated in a 12-well plate at a density of 2.5×10^5^ cells/well. When 95–100% confluency was achieved, a straight scratch was made on the surface using a pipette tip. Then, the cells were grown for 24 or 48 h in serum-free medium, and the migration of cells into the wounded area was photographed using a JULI^TM^ Smart fluorescent cell analyzer (Digital Bio source, Seoul, Korea). The wound closure rate was determined using the initial and final wound widths, and the wound closure percentage was calculated by dividing the change in wound width by the initial wound width, as described previously (40).

### Soft Agar Colony Formation Assay

Soft agar colony formation assay was performed to evaluate the anchorage-independent growth ability of the cells. Approximately 5×10^3^ cells were suspended in the top soft agar layer (0.35% soft agar) and seeded into 6-well plates, which were pre-coated with 0.5% base agar. Colonies were allowed to grow at 37°C in a 5% CO_2_ incubator for 2–3 weeks, and colony numbers were counted using an ECLIPSE Ti inverted microscope and the NIS-Elements AR (V. 4.0) computer software program (NIKON Instruments Korea, Seoul, Republic of Korea), as described previously (35).

### Correlation of CD133/NRF2 With Colorectal Cancer Prognosis

We used gene expression data of colorectal adenocarcinoma patients (n=526) available at the Cancer Genome Atlas (TCGA) Pan-Cancer Atlas data set. Analyzed data were visualized using the cBioPortal (http://cbioportal.org) to investigate the gene expression levels of NRF2, NQO1, and PIK3CA depending CD133 mRNA levels. Levels of mRNA are presented as RSEM processed using the RNA-Seq by Estimation Maximization (RSEM) algorithm in log2 scale. In addition, we examined the overall survival rates depending on CD133 expression levels and genetic alterations in *CD133* and *NRF2.* These Kaplan-Meier survival estimates were generated by log-rank nonparametric test in the cBioPortal. P-values are derived from student t-test, and q-values are obtained from Benjamini-Hochberg procedure.

### Statistical Analysis

We conducted multiple comparison tests for different treatment groups using histomorphometric analysis. The data were analyzed using one-way analysis of variance (ANOVA) followed by Tukey’s multiple comparison test to determine which pairs of groups were significantly different. Statistical analyses were conducted using GraphPad Prism 5 (GraphPad Software, Inc., La Jolla, CA, USA). Differences were considered statistically significant at P < 0.05.

## Results

### High CD133 Levels Are Associated With Facilitated Sphere Growth and CSC Marker Expression

**Figure 1:**
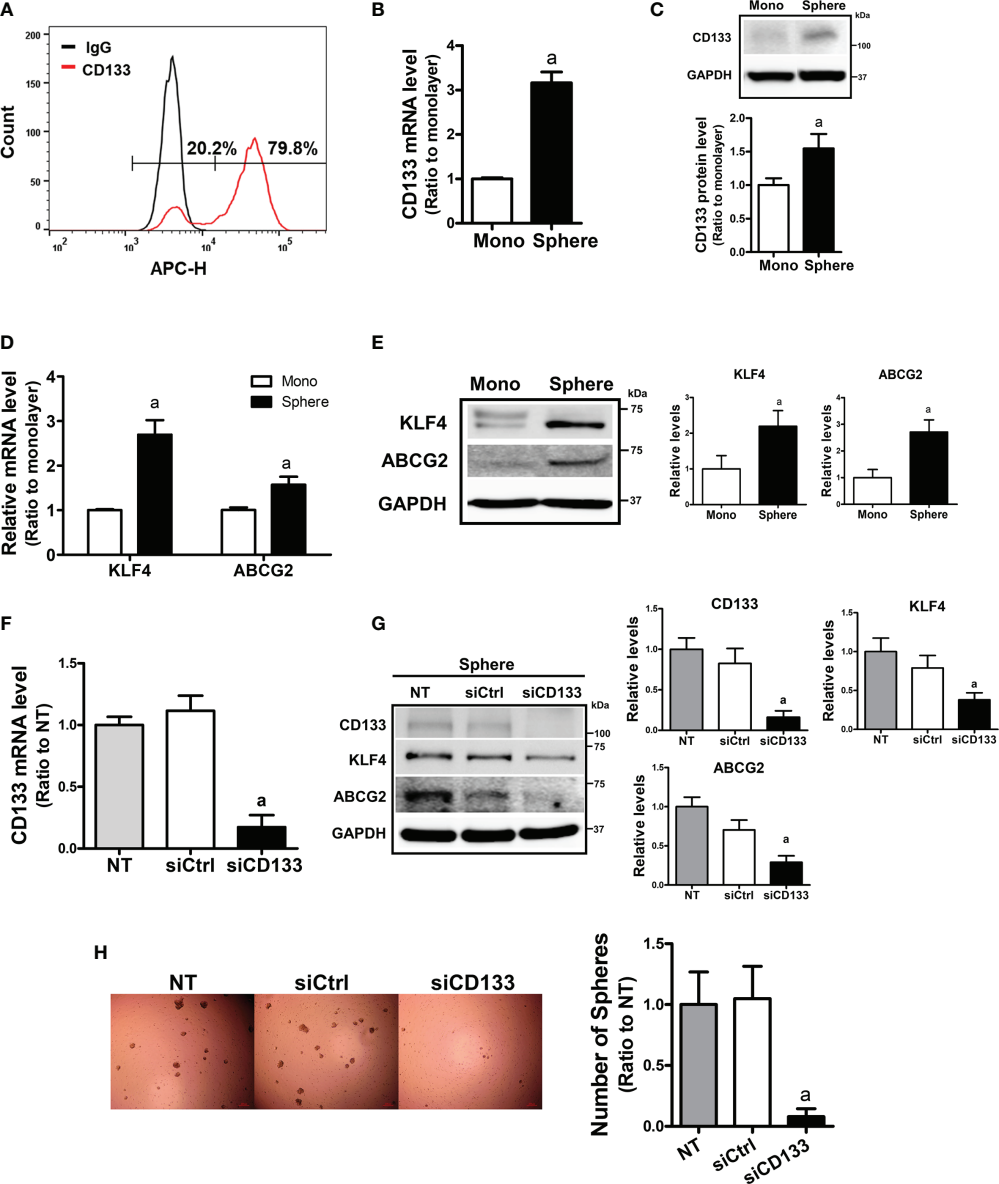
Enhanced CD133 levels in sphere cultured HCT116 cells and their involvement in sphere formation. **(A)** The presence of CD133-positive cell population in monolayer-cultured HCT116 cells was analyzed using allophycocyanin (APC)-fluorescence-based fluorescence-activated cell sorting method. **(B, C)** Transcript **(B)** and protein levels **(C)** of CD133 were assessed in monolayer and sphere cultured HCT116 cells using relative quantitative reverse transcription-polymerase chain reaction (qRT-PCR) analysis and western blotting. Bar graph represents quantified protein levels from at least three experiments. ^a^P < 0.05 compared with the monolayer HCT116. **(D, E)** The transcript **(D)** and protein levels **(E)** of Kruppel-like factor 4 (KLF4) and ATP-binding cassette subfamily G member 2 (ABCG2) in monolayer and sphere HCT116 cells were assessed. Bar graph represents quantified protein levels from at least three experiments. ^a^P < 0.05 compared with the monolayer HCT116 cells. **(F)** Transcript levels of CD133 in the non-transfected (NT), non-specific small interfering RNA (siRNA) (siCtrl)- or CD133-specific siRNA (siCD133)-transfected HCT116 cells. ^a^P < 0.05 compared with the siCtrl group. **(G)** HCT116 cell with either the non-specific siRNA (siCtrl) or CD133-specific siRNA (siCD133) transfection, were grown in sphere culture systems and the protein levels of CD133, KLF4, ABCG2 were examined. Bar graph represents quantified protein levels from at least three experiments. **(H)** Sphere formation was assessed in the non-transfected (NT), non-specific siRNA (siCtrl)- or CD133-specific siRNA (siCD133)-transfected HCT116 cells. Number of spheres over 70 μm diameter was counted using an image processing ToupView software. ^a^P < 0.05 compared with the siCtrl group. Quantification results of western blotting were relative values to the loading control GAPDH. All values represent the mean ± standard error of the mean (SEM) of more than three experiments.

To examine the relationship between CD133 and CSC-like properties, we first examined the expression levels of CD133 in different carcinoma cell lines. When the basal levels of CD133 were determined in colorectal carcinoma cell line HCT116 and Colo205, pancreatic carcinoma cell line PANC-1 and MIA PaCa-2, and breast carcinoma cell line MCF7, HCT116 showed the highest level of CD133 (**Supplementary Figures 1A, B, 2B**). FACS analysis with CD133-specific antibody showed that 79.8% of HCT116 total cell fraction expressed CD133 (**Figure 1A**). In an attempt to investigate the role of CD133 in CSC-like property development, we used a sphere culture system, which was shown to be a CSC-enriched system (42). When HCT116 cells were cultured in ultra-low attachment plates, the transcript level of CD133 increased 3.16-fold in HCT116 spheres and CD133 protein levels were also elevated (**Figures 1B, C**). Whereas, sphere culture of Colo205, PANC-1, and MIA-PaCa-2 did not show the elevations in CD133, which shows that HCT116 can be used as our experimental model (**Supplementary Figures 2A, B**). In accordance with CD133 elevation, levels of CSC markers, including KLF4 and ABCG2, were significantly increased in HCT116 colonospheres (**Figures 1D, E**). We then assessed the association of CD133 with CSC-like properties by silencing *CD133* in HCT116 (**Figure 1F**). The *CD133*–silenced colonospheres expressed lower levels of KLF4 and ABCG2 than the nonspecific siRNA-transfected colonospheres (**Figure 1G**). In addition, the number of spheres with a diameter greater than 70 μm was markedly diminished by *CD133* silencing (**Figure 1H**). These results showed the critical contribution of CD133 to the development of CSC-like properties in colon cancer cells.

### NRF2 Elevation Is Mediated by CD133 in Sphere Cultured Colon Cancer Cells

**Figure 2:**
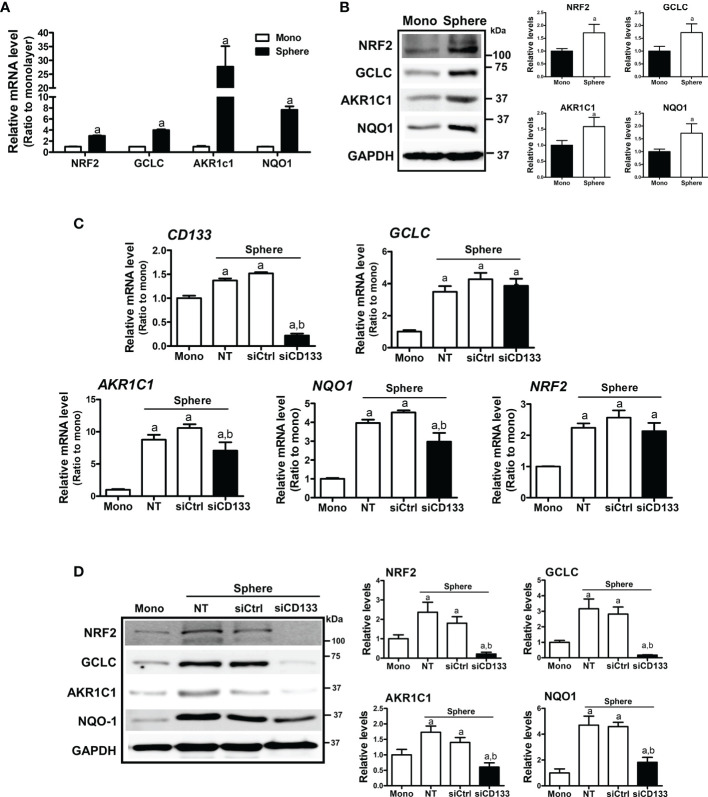
Association of CD133 with NRF2 upregulation in HCT116 spheres. **(A)** Transcript levels of *NRF2*, *GCLC*, *AKR1C1*, and *NQO1* were measured in monolayer and sphere HCT116 cells using a relative qRT-PCR analysis. ^a^P < 0.05 compared with the monolayer group. **(B)** Protein levels of NRF2, GCLC, AKR1C1 and NQO1 were assessed in monolayer and sphere HCT116 cells. Bar graph represents quantified protein levels from at least three experiments. ^a^P < 0.05 compared with the monolayer group. **(C)** HCT116 with either the non-transfection (NT), non-specific siRNA (siCtrl) or CD133-specific siRNA (siCD133) transfection were grown in the sphere culture system, and transcript levels of *CD133*, *GCLC*, *AKR1C1*, *NQO1*, and *NRF2* were measured. ^a^P < 0.05 compared with the monolayer. ^b^P < 0.05 compared with the siCtrl group. **(D)** Protein levels for NRF2, GCLC, AKR1C1, and NQO1 were monitored in sphere cultured HCT116 cells with either the non-specific siRNA (siCtrl) or CD133-specific siRNA (siCD133) transfection. Bar graph represents quantified protein levels from at least three experiments. Quantification results of western blotting were relative values to the loading control GAPDH. ^a^P < 0.05 compared with the monolayer. ^b^P < 0.05 compared with the siCtrl group. NT, non-transfection group.

In HCT116 colonospheres, transcription levels of NRF2 and its target genes, such as *GCLC*, *AKR1C1*, and *NQO1* were all increased compared to the HCT116 monolayer (**Figure 2A**). Increased protein levels of NRF2, GCLC, AKR1C1, and NQO1 were also confirmed by western blotting (**Figure 2B**). Next, we tested the potential involvement of CD133 in NRF2 signaling activation by silencing *CD133* in colonospheres. When *CD133*-silenced HCT116 was cultured in sphere conditions, the elevations in NRF2 and its targets GCLC, AKR1C1, and NQO1 were attenuated when compared to the control siRNA-transfected spheres (**Figures 2C, D**). Whereas, NRF2 mRNA levels, which were elevated in spheres, were not significantly reduced by *CD133* silencing. These results showed that the high NRF2 level in sphere-cultured HCT116 cells was attributed to CD133.

### Activation of PI3K/AKT Signaling Is Involved in CD133-Mediated Elevation of NRF2

**Figure 3:**
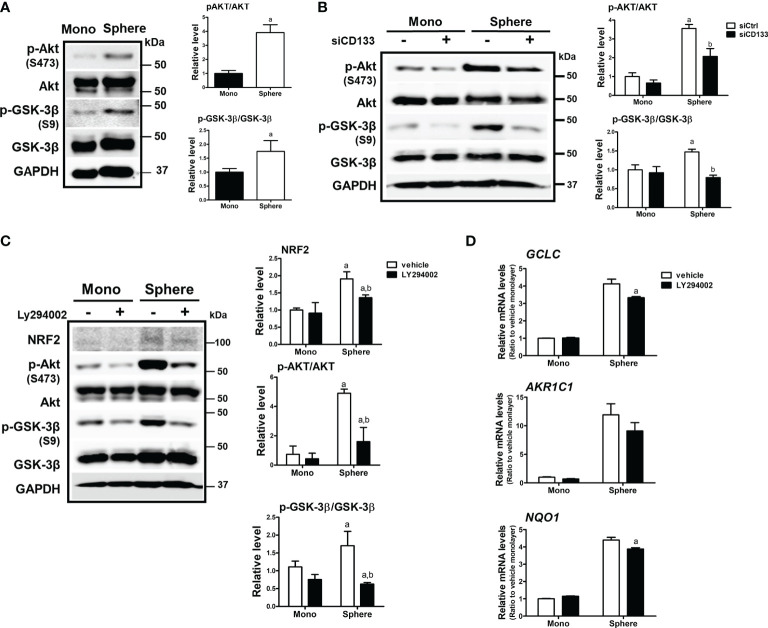
Involvement of PI3K/AKT/GSK-3β signaling in NRF2 activation in HCT116 spheres. **(A)** Protein levels of p-AKT (S473), total AKT, p-GSK-3β (S9), and total GSK-3β were determined in monolayer and sphere HCT116 cells. Bar graph represents quantified protein levels from at least three experiments. ^a^P < 0.05 compared with the monolayer group. **(B)** HCT116 cells were transfected with the non-specific siRNA (siCtrl) or CD133-specific siRNA (siCD133) and grown in either monolayer or sphere culture system. Protein levels for p-AKT (S473), AKT, p-GSK-3β (S9), and GSK-3β were assessed using western blotting. Bar graph represents quantified protein levels from at least three experiments. ^a^P < 0.05 compared with the monolayer group. **(C)** LY294002 (10 μM), a pharmacological inhibitor of PI3K, was incubated in monolayer and sphere HCT116 cells for 24 h, and protein levels of NRF2, p-AKT (S473), AKT, p-GSK-3β (S9), and GSK-3β were determined. Bar graph represents quantified protein levels from at least three experiments. ^a^P < 0.05 compared with the monolayer vehicle group. ^b^P < 0.05 compared with the sphere vehicle group. **(D)** Transcript levels of *GCLC AKR1C1*, and *NQO1* were measured using a relative qRT-PCR analysis in vehicle- or LY294002-treated cells. ^a^P < 0.05 compared with the vehicle-treated sphere group. Quantification results of western blotting were relative values to the loading control GAPDH. Values represent the mean ± SEM of more than three experiments.

There have been reports showing that CD133 induces PI3K/AKT signaling activation in colon cancer cells as well as glioma stem cells (43, 44). In addition, AKT-mediated GSK-3β phosphorylation stabilizes NRF2 (27). Based on these results, we monitored the activation levels of AKT/GSK-3β using western blotting to elucidate the molecular events involved in CD133-mediated NRF2 elevation in colonospheres. The level of phosphorylated AKT (p-AKT) at Ser473 was higher in colonospheres than in monolayer HCT116 cells, implying the activation of PI3K (**Figure 3A**). Subsequently, the level of phosphorylated GSK-3β (Ser9), an inactive form of proteasomal NRF2 degradation, was elevated in sphere-cultured HCT116 cells. These results show that CD133-mediated PI3K/AKT activation and resultant GSK-3β inactivation could be a cause of NRF2 elevation in colonospheres. Indeed, when *CD133* was silenced, levels of p-AKT and p-GSK-3β were reduced in colonospheres (**Figure 3B**), and treatment with the PI3K inhibitor LY294002 (10 μM) repressed these elevations (**Figure 3C**). Additionally, PI3K inhibitor treatment diminished NRF2 levels and target gene expression levels in HCT116 spheres (**Figures 3C, D**). These results showed that CD133 contributes to NRF2 upregulation *via* PI3K/AKT activation and subsequent inhibition of p-GSK-3β.

### NRF2 Contributes to Facilitated Sphere Formation and CSC Marker Elevation

**Figure 4:**
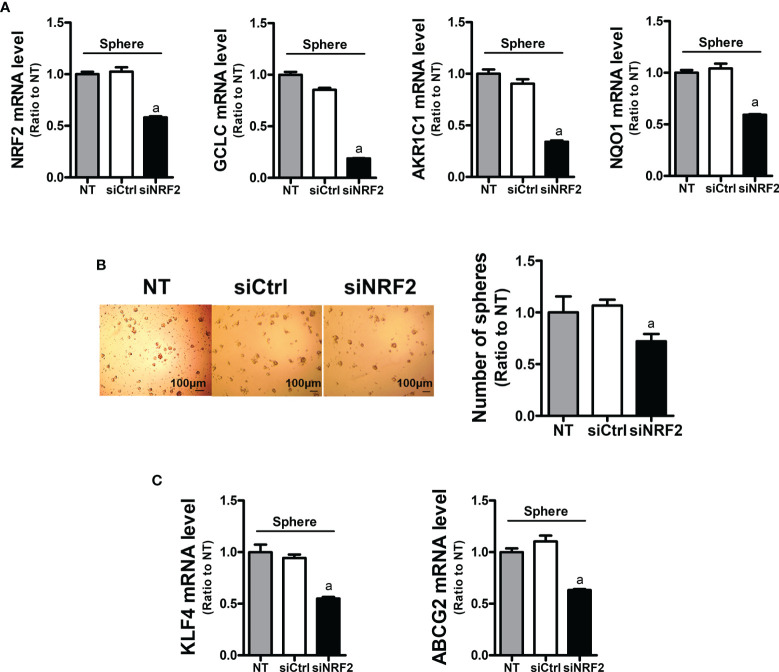
Inhibition of sphere formation and CSC marker levels in *NRF2*-silenced HCT116 cells. **(A)** HCT116 cells were transfected with the non-specific siRNA (siCtrl) or *NRF2*-specific siRNA (siNRF2) and grown for 3 days in sphere culture condition. Levels of *NRF2, GCLC, AKR1C1,* and *NQO1* mRNAs were determined in the siCtrl and siNRF2 spheres. ^a^P < 0.05 compared to the siCtrl group. **(B)** The siCtrl and siNRF2 HCT116 were grown in sphere culture condition, and sphere formation was assessed. Number of spheres over 70 μm diameter was counted using an image processing ToupView software. Values represent the mean ± SEM from three independent experiments. ^a^P < 0.05 compared to the siCtrl group. **(C)** Levels of *KLF4* and *ABCG2* mRNA were assessed in the siCtrl and siNRF2 spheres. ^a^P < 0.05 compared to the siCtrl group. All values in RT-PCR analysis represent the mean ± SEM from at least three independent experiments. NT, non-transfection group.

In order to assess the role of NRF2 in colonosphere formation, *NRF2*-silenced HCT116 was cultured in sphere culture condition (**Figure 4A**). *NRF2*-silencing affected the sphere-forming capacity of HCT116 cells, and the sphere number was reduced (**Figure 4B**). In line with this, the levels of CSC markers KLF4 and ABCG2 were significantly lower in the *NRF2*-silenced colonospheres than in the control siRNA-transfected colonospheres (**Figure 4C**). These results confirmed the functional contribution of NRF2 to the sphere-forming capacity.

### CD133^high^ HCT116 Cells Display Enhanced CSC-Like Properties and NRF2 Activation

**Figure 5:**
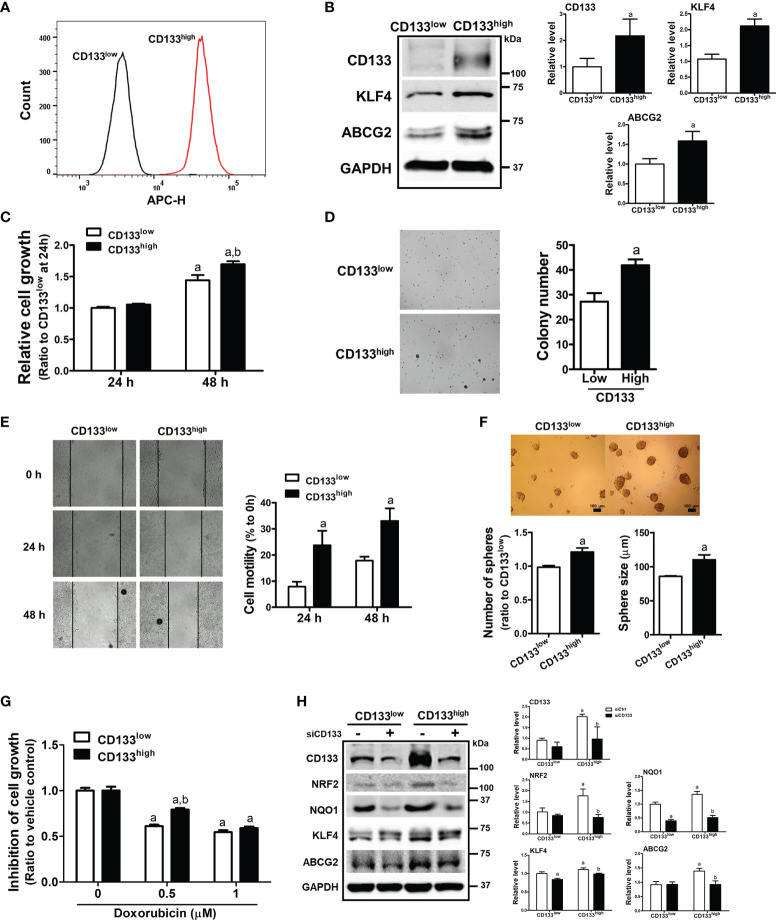
Enhanced CSC-like properties and NRF2 levels in CD133^high^ HCT116 fraction. **(A)** CD133-positive and CD133-negative cell fractions were isolated using FACS analysis, and the CD133^high^ and CD133^low^ HCT116 cell lines were established. CD133-positive cell fraction in the CD133^high^ and CD133^low^ HCT116 cell lines was assessed following maintenance for 2 weeks. **(B)** Protein levels of CD133, KLF4, and ABCG2 were determined in established CD133^high^ and CD133^low^ HCT116 cells. Bar graph represents quantified protein levels from at least three experiments. ^a^P < 0.05 compared with the CD133^low^ cell line. **(C)** The CD133^high^ and CD133^low^ HCT116 cells were grown in the absence of fetal bovine serum (FBS) and relative cell growth was assessed using MTT assay after 24 h and 48 h of plating. ^a^P < 0.05 compared to each 24 h group. ^b^P < 0.05 compared to CD133^low^ HCT116 cells. **(D)** The CD133^high^ and CD133^low^ HCT116 cells were suspended in the top soft agar layer (0.35% soft agar) and anchorage-independent growth was monitored for 2 weeks. Colony number was counted using an ECLIPSE Ti inverted microscope and the NIS-Elements AR (V. 4.0) software. Bar graph represents quantified results from at least three experiments. ^a^P < 0.05 compared to the CD133^low^ HCT116 cells. **(E)** Cell migration ability was assessed in the CD133^high^ and CD133^low^ HCT116 cells using a wound-healing assay for 24 h and 48 h. Bar graph represents quantified results from at least three experiments. ^a^P < 0.05 compared to the CD133^low^ HCT116 cells. **(F)** CD133^high^ and CD133^low^ HCT116 cells were grown in sphere culture condition, and sphere formation capacity was assessed by measuring the number and average size of the spheres. Bar graph represents quantified results from at least three experiments. ^a^P < 0.05 compared to the CD133^low^ HCT116 cells. **(G)** Inhibition of cell growth was monitored following doxorubicin (0.5 and 1 μM) incubation for 24 h using MTT assay. Values represent the mean ± SEM from four independent experiments. ^a^P < 0.05 compared to the doxorubicin-treated CD133^low^ group. **(H)** CD133^high^ and CD133^low^ HCT116 cells were transfected with *CD133* siRNA and protein levels of CD133, NRF2, NQO1, KLF4, and ABCG2 were determined. Bar graph represents quantified protein levels from at least three experiments. Quantification results of western blotting were relative values to the loading control GAPDH. All values represent the mean ± SEM from three independent experiments. ^a^P < 0.05 compared with the CD133^low^ siCtrl group. ^b^P < 0.05 compared with the CD133^high^ siCtrl group.

To confirm the relationship between CD133/NRF2 and CSC-like properties, we isolated CD133-positive and CD133-negative cell fractions from total HCT116 cells, and established CD133^low^ and CD133^high^ cell lines (**Figure 5A**). These cell lines were cultured for up to 1 month, and high CD133 expression was maintained along with elevated ABCG2 and KLF4 levels (**Figure 5B**). As phenotypic characteristics, the cell growth rate of CD133^high^ HCT116 cells was higher than that of CD133^low^ cells (**Figure 5C**), and anchorage-independent colony formation was enhanced (**Figure 5D**). In addition, cell motility in the wound healing assay (**Figure 5E**) and sphere forming capacity (**Figure 5F**) were higher in CD133^high^ HCT116 cells than in CD133^low^ cells. In line with these CSC-like properties, the cytotoxic response to anticancer treatment was determined using doxorubicin. We observed that 0.5 to 1 μM doxorubicin treatment for 24 h showed similar rates of growth inhibition, and the cell growth inhibition by 0.5 μM doxorubicin treatment was alleviated in CD133^high^ HCT116 cells (**Figure 5G**). When CD133 levels were silenced in CD133^high^ HCT116 cells, the levels of the CSC markers ABCG2 and KLF4, which were elevated in this cell line, were diminished (**Figure 5H**). Additionally, the levels of NRF2 and NQO1 were higher in CD133^high^ HCT116 cells than in CD133^low^ cells and were repressed by *CD133* silencing (**Figure 5H**). These results provide direct evidence that the CD133-enriched population exhibited enhanced colony formation, cell migration, colony formation, and drug resistance, which is accompanied by NRF2 activation.

### CD133 Alterations Are Associated With NRF2 Elevation and Poor Clinical Outcome in Patients With Colorectal Cancer

**Figure 6:**
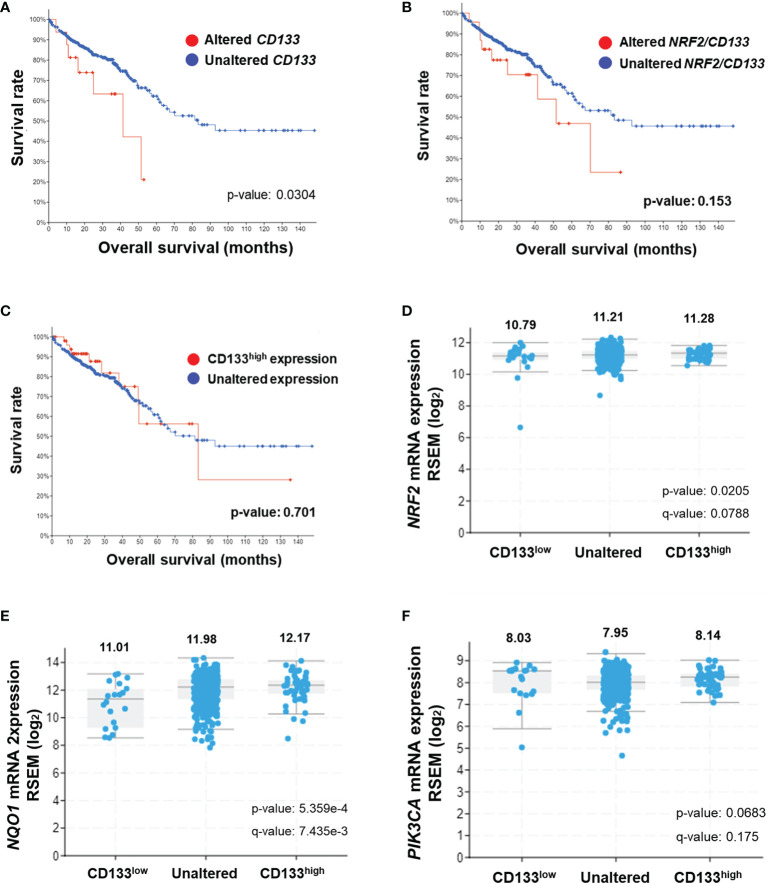
Association of CD133 with NRF2 and clinical outcome of colorectal cancer patients. **(A)** Overall survival rates of colorectal patients with altered (n = 17) *vs* unaltered (n = 509) *CD133* gene. **(B)** Overall survival rates of colorectal patients with altered (n = 24) vs unaltered (n = 502) *NRF2* or *CD133* gene. **(C)** Overall survival rates of colorectal patients with high CD133 (n = 52) *vs* unaltered (474) mRNA levels. Plots generated by cBioPortal were modified. **(D–F)** Correlation of CD133 mRNA levels (high CD133, n = 52; unaltered CD133, n = 454; low CD133, n = 20) with NRF2 **(D)**, NQO1 **(E)**, and PIK3CA **(F)** mRNA levels in colorectal tumors. Values are given in (RNA Seq V2 RSEM) in log2. Plots generated by cBioPortal were modified.

In an attempt to investigate the clinical implication of the linkage between CD133 and NRF2, we analyzed clinical data from the TCGA Pan-Cancer Atlas database using the cBioPortal interface. A total of 526 gene expression data from colorectal adenocarcinoma patients were available in the TCGA Pan-Cancer Atlas database, and 17 cases showed genetic alterations in *CD133* gene (missense mutation, 15; frameshift deletion, 1; splice, 1). In *CD133*-altered group, 11.8% of patient samples demonstrated *NRF2* gene alteration, whereas only 1.4% of samples showed *NRF2* alteration in *CD133*-unaltered group (data not shown). First, the clinical relationship between *CD133* gene alteration (n=17) and colorectal cancer patients survival was assessed by Kaplan-Meier estimate analysis. It revealed shorter overall survival rate in patients with altered *CD133* gene (median survival months=41.36) compared to unaltered *CD133* patients (median survival months=83.24) (**Figure 6A**). Additionally, patients whose tumors have *NRF2* or *CD133* gene alteration showed shorter overall survival estimates (median survival months=51.48) than patients with unaltered *CD133* gene groups (median survival months=83.24) (**Figure 6B**). These imply the potential correlation between *CD133* gene alteration and clinical outcome of colorectal cancer patients.

Among 526 colorectal cancer patients, 52 patients exhibited higher *CD133* mRNA levels when compared to unaltered CD133 mRNA group (n=474). The Kaplan-Meier survival analysis showed that the median survival month of patients with high CD133 expression is 81.37 month, which is lower than that of patients with unaltered CD133 expression (83.24 month), although no statistical significance obtained (**Figure 6C**). In addition, CD133 mRNA levels were associated with increased mRNA levels of NRF2 and NQO1 in these patients. Mean log2 mRNA expressions of NRF2 were 10.79, 11.21, and 11.28 in CD133-low, unaltered, and CD133-high group, respectively (**Figure 6D**). NQO1 levels were increased depending on CD133 levels (**Figure 6D**). PIK3CA mRNA levels were also relatively high in CD133-high group when compared to unaltered group (**Figure 6F**). These results indicate that CD133 expression is associated with NRF2 signaling activation in colorectal cancers, and further suggested the correlation of CD133 alteration (mutation and increased expression) with unfavorable clinical outcome.

## Discussion

The major characteristics of CSCs, which include refractory response to conventional chemotherapy and radiotherapy, can be explained by the elevation of drug efflux transporters, enhanced DNA repair ability, and activation of the ROS defense system. The side population of cancer cells, which excludes the fluorescent dye Hoechst 33342, expresses a high level of ABCG2 (BCRP) and displays chemoresistant phenotypes (45). The CD133-high fraction from glioma cells was more resistant to radiotherapy than the CD133-negative fraction, and CD133-high cells activated the molecular event for the DNA damage checkpoint and enhanced DNA repair capacity following radiotherapy (46). The CD44^+^/CD24^-^ cell fraction was found to be resistant to radiation, and maintenance of low ROS levels was associated with radioresistance (47). CSCs from human breast tumors exhibited less DNA damage and a higher rate of survival after irradiation compared to non-CSCs, and low levels of ROS in CSCs were attributed to increased expression levels of ROS detoxifying systems, such as glutamate-cysteine ligase and GSH synthetase (9). In leukemia with a high frequency of stem cells, ROS levels were low and ROS-scavenging GPX3 levels were high compared to leukemia with a low frequency of stem cells (48). Leukemic stem cells exhibited high levels of FoxoO3a expression, and deletion of foxO blocked the initiation of myeloid leukemia in a mouse model (49). In head and neck squamous cell carcinoma, a CSC marker CD44 variant was found to directly bind to the cystine/glutamate antiporter xCT, thereby increasing GSH synthesis, which is involved in therapy resistance (50). These reports consistently support the importance of the ROS detoxifying system in CSCs for maintaining low ROS levels and high survival under therapy stress.

In the current study, we demonstrated a positive linkage between CD133 and NRF2 in CSC-like properties using the colonosphere culture system and the CD133^high^ subpopulation. Sphere culture of the colorectal cell line HCT116 led to an increase in CD133 expression along with elevated CSC markers, such as KLF4 and ABCG2. *CD133*-silencing suppressed sphere-forming capacity and expression of KLF4 and ABCG2, which indicates the critical role of CD133 in CSC-like properties development. NRF2 signaling was also upregulated in colonospheres and partly responsible for CSC marker elevation and sphere-forming capacity. Notably, NRF2 activation in colonospheres was CD133-dependent: *CD133*-silencing inhibited NRF2 elevation and attenuated the expression of GCLC, AKR1C1, and NQO1. The relationship between CD133 and NRF2 was confirmed in an isolated CD133^high^ subpopulation from HCT116 cells. Established CD133^high^ cell line showed higher doxorubicin resistance, colony formation, sphere formation, and migration capacity than CD133^low^ cell line, and *CD133*-silencing in CD133^high^ cells suppressed NRF2 activation and KLF4 elevation. These *in vitro* results were supported by clinical relationship between CD133 and NRF2, which were obtained from ATCG database. In colorectal carcinoma patients, overall survival rates were diminished by *CD133* and *CD133/NRF2* gene alterations, and high CD133 mRNA levels also showed a relationship with reduced overall survival rates. Additionally, high CD133 mRNA levels in colorectal carcinoma showed positive correlations with high transcript levels of NRF2 and NQO1. Taken together, these results suggest that CD133 mediates the activation of NRF2 signaling, which in turn contributes to the CSC-like properties of CD133^high^ colon cancers.

As a molecular event for CD133-mediated NRF2 activation, we suggest the involvement of PI3K/AKT/GSK-3β. Multiple reports have suggested the activation and contribution of PI3K/AKT signaling in CD133-positive CSCs. Microarray analysis revealed that expression of genes related to the PI3K/AKT signaling pathway was elevated in sphere-cultured CD133^+^/CD44^-^ prostate CSCs, and knockdown of phosphatase and tensin homolog (PTEN) stimulated sphere formation by inhibiting PI3K/AKT signaling (51). Phosphorylation of Tyr828 residue in the cytoplasmic domain of CD133 mediates binding with PI3K, and subsequently activates PI3K/AKT signaling for self-renewal and tumorigenicity of glioma stem cells (44). In line with these findings, an inhibitor of PI3K/AKT suppressed the proliferation and stemness of colon CSCs (52). In our colonosphere system, activation of the PI3K/AKT axis and consequent phosphorylation of GSK-3β at Ser9 were observed. Since AKT-mediated phosphorylation inhibits GSK-3β activity for β-TCRP-dependent degradation of NRF2, activated PI3K/AKT signaling is often associated with NRF2 activation in multiple types of cancers. In breast cancers with oncogenic PI3K/AKT activation, NRF2-driven GSH biosynthesis is stimulated, which is required for oxidative stress resistance, tumor spheroid formation, and colony formation. In addition, elevation of NRF2 targets showed a positive correlation with mutation status in the PI3K/AKT pathway (53). In the absence of *KEAP1*, the deletion of *PTEN* could further elevate NRF2 levels, which accompanied GSK-3β inactivation *via* PI3K/AKT activation (8, 54). In our study with colonospheres, *CD133*-silencing reduced phosphorylation of AKT/GSK-3β, and the PI3K inhibitor LY294002 blocked AKT-mediated GSK-3β phosphorylation and attenuated NRF2 target genes expression. These results suggested that CD133 activates PI3K/AKT signaling, which in turn stabilizes NRF2 protein *via* GSK-3β inhibition in colonospheres. Of note, we observed that transcript levels of NRF2 were also higher in colonospheres than those in monolayer cultured cells (**Figures 2A, C**). As NRF2 transcription is regulated by its 5-flanking upstream ARE as a positive feedback loop (55), it can be plausible that PI3K/AKT-mediated NRF2 stabilization elevates NRF2 transcription.

Several reports have demonstrated that NRF2 signaling plays a role in CSC maintenance and therapy resistance. In primary glioma stem cells from human glioblastoma tissues, NRF2 knockdown disrupts self-renewal and pluripotency (38). NRF2 signaling is elevated in spheroid cultured breast cancer cells, and high NRF2 levels are required for the maintenance of low ROS levels and taxol resistance (56). Similarly, NRF2 elevation in mammospheres resulted in high levels of drug efflux transporters and antioxidant genes, and NRF2-silencing blocked sphere growth and induced chemosensitization (37). Approximately 3.1% of cervical CSCs were isolated from tumor specimens, NRF2 was aberrantly upregulated, and NRF2 silencing could sensitize cervical CSCs to DNA damage-induced apoptosis (34). CD133^+^/CD44^+^ colon CSCs express high levels of ABCB1 *via* NRF2 elevation, which is associated with doxorubicin resistance (57). In our previous study, CD44^high^CD24^low^ breast CSCs exhibited high levels of NRF2 signaling, and NRF2-silencing led to retarded tumor growth, suppression of sphere formation and invasion capacity, and anticancer sensitization (36). The ovarian CSC fraction with high ALDH1 retained low levels of ROS, which was accompanied by NRF2 signaling activation (35). These results suggested a critical role of NRF2 signaling in CSC maintenance and tumor resistance/recurrence and support our current observation of the association between CD133 and NRF2.

**Figure 7:**
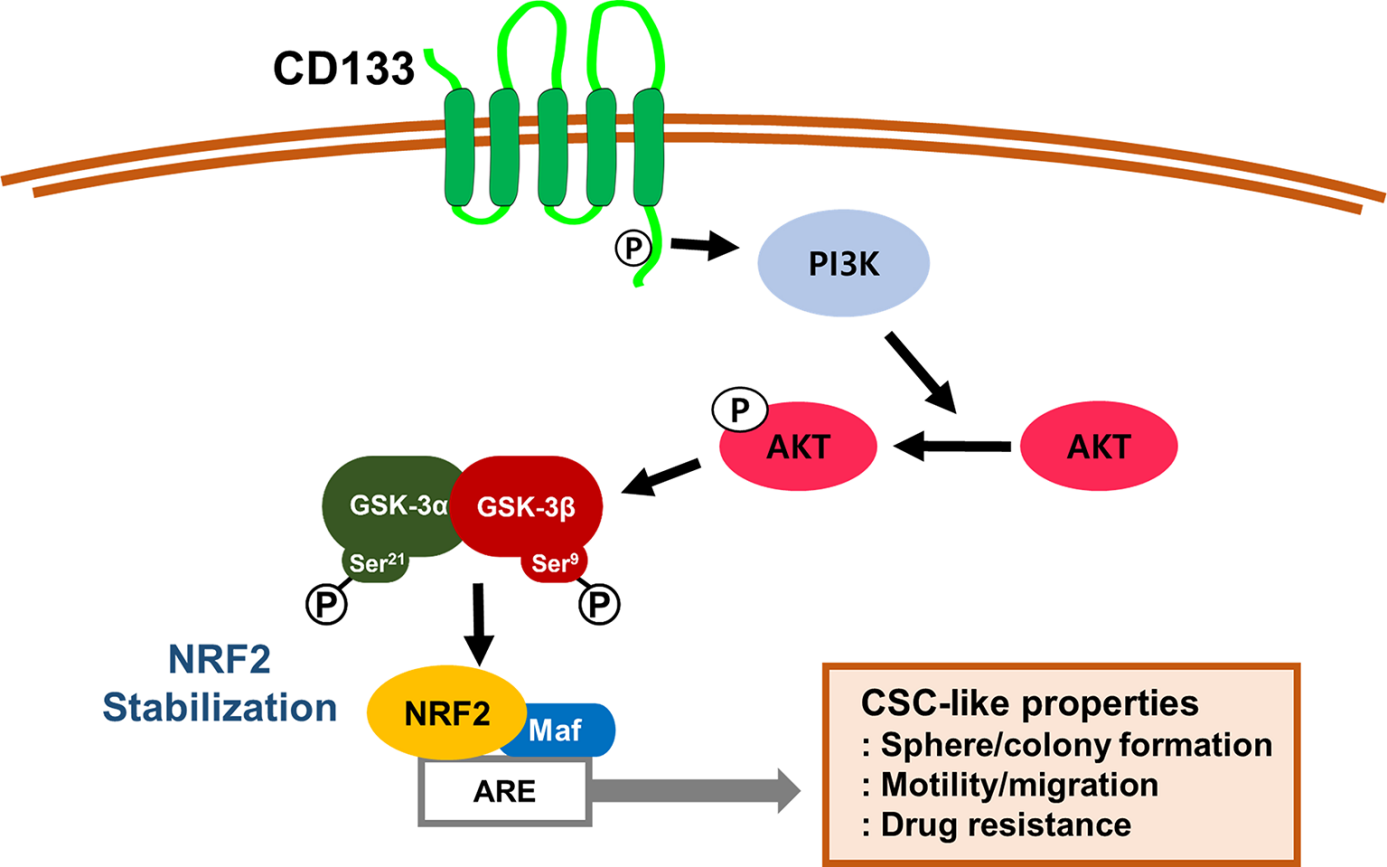
A schematic diagram of the activation of NRF2 signaling and the development of CSC-like properties in CD133-enriched cancer cells. In colonospheres and the CD133-positive cell fraction, high CD133 levels induce PI3K/AKT activation and subsequently inactivate GSK-3β to stabilize NRF2 protein, which results in the acquisition of CSC-like properties, including anchorage-independent growth, sphere formation, facilitated migration, and resistance to anticancer drugs.

Taken together, our results indicate that CD133, a molecular marker of colon CSCs, leads to PI3K/AKT-associated NRF2 activation (**Figure 7**). High NRF2 levels in spheroid cultured HCT116 cells and the CD133^high^ subpopulation contributed to the aggressive CSC phenotypes, including anticancer resistance, sphere formation, anchorage-independent colony formation, and migration potential. Therefore, the NRF2 axis might be a promising target for the inhibition of therapeutic resistance and enhancement of survival capacity under stress conditions in CD133^high^ CSCs.

## Data Availability Statement

The original contributions presented in the study are included in the article/**Supplementary Material**. Further inquiries can be directed to the corresponding author.

## Author Contributions

JP carried out the experiment with support from SKK and SPH. SKK and SPH contributed to sample preparation, data analysis, and figure preparation. JP and MKK wrote the manuscript with the help from SKK and SPH. BHC contributed to data analysis and figure preparation. MKK conceived the original idea and supervised the project. All authors provided critical feedback and helped shape the research, analysis and manuscript preparation. All authors contributed to the article and approved the submitted version.

## Funding

This work was supported by the National Research Foundation of Korea (NRF) grant funded by the Korean government (MSIT) (2018R1A2A1A05078894, 2018R1A6A1A03025108).

## Conflict of Interest

The authors declare that the research was conducted in the absence of any commercial or financial relationships that could be construed as a potential conflict of interest.

## Publisher’s Note

All claims expressed in this article are solely those of the authors and do not necessarily represent those of their affiliated organizations, or those of the publisher, the editors and the reviewers. Any product that may be evaluated in this article, or claim that may be made by its manufacturer, is not guaranteed or endorsed by the publisher.

## References

[1] Bonnet D Dick JE. Human Acute Myeloid Leukemia is Organized as a Hierarchy That Originates From a Primitive Hematopoietic Cell. Nat Med (1997) 3(7):730–7. doi: 10.1038/nm0797-730 9212098

[2] Huang R Rofstad EK. Cancer Stem Cells (CSCs), Cervical CSCs and Targeted Therapies. Oncotarget (2017) 8(21):35351–67. doi: 10.18632/oncotarget.10169 PMC5471060 27343550

[3] Chen W Dong J Haiech J Kilhoffer M-C Zeniou M. Cancer Stem Cell Quiescence and Plasticity as Major Challenges in Cancer Therapy. Stem Cells Int (2016) 2016:1740936–. doi: 10.1155/2016/1740936 PMC4932171 27418931

[4] Shahriyari L Komarova NL. Symmetric vs. Asymmetric Stem Cell Divisions: An Adaptation Against Cancer? PloS One (2013) 8(10):e76195. doi: 10.1371/journal.pone.0076195 24204602 PMC3812169

[5] Khan AQ Ahmed EI Elareer NR Junejo K Steinhoff M Uddin S. Role of miRNA-Regulated Cancer Stem Cells in the Pathogenesis of Human Malignancies. Cells (2019) 8(8):840. doi: 10.3390/cells8080840 PMC6721829 31530793

[6] Zhao J. Cancer Stem Cells and Chemoresistance: The Smartest Survives the Raid. Pharmacol Ther (2016) 160:145–58. doi: 10.1016/j.pharmthera.2016.02.008 PMC4808328 26899500

[7] Tan BT Park CY Ailles LE Weissman IL. The Cancer Stem Cell Hypothesis: A Work in Progress. Lab Invest (2006) 86(12):1203–7. doi: 10.1038/labinvest.3700488 17075578

[8] Abdullah LN Chow EK-H. Mechanisms of Chemoresistance in Cancer Stem Cells. Clin Trans Med (2013) 2(1):3–. doi: 10.1186/2001-1326-2-3 PMC3565873 23369605

[9] Diehn M Cho RW Lobo NA Kalisky T Dorie MJ Kulp AN. Association of Reactive Oxygen Species Levels and Radioresistance in Cancer Stem Cells. Nature (2009) 458(7239):780–3. doi: 10.1038/nature07733 PMC2778612 19194462

[10] Villodre ES Kipper FC Pereira MB Lenz G. Roles of OCT4 in Tumorigenesis, Cancer Therapy Resistance and Prognosis. Cancer Treat Rev (2016) 51:1–9. doi: 10.1016/j.ctrv.2016.10.003 27788386

[11] Yu F Li J Chen H Fu J Ray S Huang S. Kruppel-Like Factor 4 (KLF4) is Required for Maintenance of Breast Cancer Stem Cells and for Cell Migration and Invasion. Oncogene (2011) 30(18):2161–72. doi: 10.1038/onc.2010.591 PMC3088782 21242971

[12] Weigmann A Corbeil D Hellwig A Huttner WB. Prominin, a Novel Microvilli-Specific Polytopic Membrane Protein of the Apical Surface of Epithelial Cells, is Targeted to Plasmalemmal Protrusions of non-Epithelial Cells. Proc Natl Acad Sci USA (1997) 94(23):12425–30. doi: 10.1073/pnas.94.23.12425 PMC24979 9356465

[13] Miraglia S Godfrey W Yin AH Atkins K Warnke R Holden JT. A Novel Five-Transmembrane Hematopoietic Stem Cell Antigen: Isolation, Characterization, and Molecular Cloning. Blood (1997) 90(12):5013–21. doi: 10.1182/blood.V90.12.5013 9389721

[14] Aghajani M Mansoori B Mohammadi A Asadzadeh Z Baradaran B. New Emerging Roles of CD133 in Cancer Stem Cell: Signaling Pathway and miRNA Regulation. J Cell Physiol (2019) 234(12):21642–61. doi: 10.1002/jcp.28824 31102292

[15] Ricci-Vitiani L Lombardi DG Pilozzi E Biffoni M Todaro M Peschle C. Identification and Expansion of Human Colon-Cancer-Initiating Cells. Nature (2007) 445(7123):111–5. doi: 10.1038/nature05384 17122771

[16] O’Brien CA Pollett A Gallinger S Dick JE. A Human Colon Cancer Cell Capable of Initiating Tumour Growth in Immunodeficient Mice. Nature (2007) 445(7123):106–10. doi: 10.1038/nature05372 17122772

[17] Horst D Kriegl L Engel J Kirchner T Jung A. CD133 Expression is an Independent Prognostic Marker for Low Survival in Colorectal Cancer. Br J Cancer (2008) 99(8):1285–9. doi: 10.1038/sj.bjc.6604664 PMC2570510 18781171

[18] Nomura A Banerjee S Chugh R Dudeja V Yamamoto M Vickers SM. CD133 Initiates Tumors, Induces Epithelial-Mesenchymal Transition and Increases Metastasis in Pancreatic Cancer. Oncotarget (2015) 6(10):8313–22. doi: 10.18632/oncotarget.3228 PMC4480754 25829252

[19] Bertolini G Roz L Perego P Tortoreto M Fontanella E Gatti L. Highly Tumorigenic Lung Cancer CD133+ Cells Display Stem-Like Features and are Spared by Cisplatin Treatment. Proc Natl Acad Sci USA (2009) 106(38):16281–6. doi: 10.1073/pnas.0905653106 PMC2741477 19805294

[20] Ma L Liu T Jin Y Wei J Yang Y Zhang H. ABCG2 is Required for Self-Renewal and Chemoresistance of CD133-Positive Human Colorectal Cancer Cells. Tumour Biol (2016) 37(9):12889–96. doi: 10.1007/s13277-016-5209-5 27449042

[21] Baird L Yamamoto M. The Molecular Mechanisms Regulating the KEAP1-NRF2 Pathway. Mol Cell Biol (2020) 40(13):e00099–20. doi: 10.1128/mcb.00099-20 32284348 PMC7296212

[22] Cho HY Kleeberger SR. Mitochondrial Biology in Airway Pathogenesis and the Role of NRF2. Arch Pharm Res (2020) 43(3):297–320. doi: 10.1007/s12272-019-01182-5 31486024 PMC7054182

[23] Otsuki A Yamamoto M. Cis-Element Architecture of Nrf2-Smaf Heterodimer Binding Sites and its Relation to Diseases. Arch Pharm Res (2020) 43(3):275–85. doi: 10.1007/s12272-019-01193-2 31792803

[24] Hayes JD Dinkova-Kostova AT. The Nrf2 Regulatory Network Provides an Interface Between Redox and Intermediary Metabolism. Trends Biochem Sci (2014) 39(4):199–218. doi: 10.1016/j.tibs.2014.02.002 24647116

[25] Shaw P Chattopadhyay A. Nrf2-ARE Signaling in Cellular Protection: Mechanism of Action and the Regulatory Mechanisms. J Cell Physiol (2020) 235(4):3119–30. doi: 10.1002/jcp.29219 PMC13482834 31549397

[26] Cuadrado A Rojo AI Wells G Hayes JD Cousin SP Rumsey WL. Therapeutic Targeting of the NRF2 and KEAP1 Partnership in Chronic Diseases. Nat Rev Drug Discov (2019) 18(4):295–317. doi: 10.1038/s41573-018-0008-x 30610225

[27] Chowdhry S Zhang Y McMahon M Sutherland C Cuadrado A Hayes JD. Nrf2 is Controlled by Two Distinct Beta-TrCP Recognition Motifs in its Neh6 Domain, One of Which can be Modulated by GSK-3 Activity. Oncogene (2013) 32(32):3765–81. doi: 10.1038/onc.2012.388 PMC3522573 22964642

[28] Salazar M Rojo AI Velasco D de Sagarra RM Cuadrado A. Glycogen Synthase Kinase-3β Inhibits the Xenobiotic and Antioxidant Cell Response by Direct Phosphorylation and Nuclear Exclusion of the Transcription Factor Nrf2. J Biol Chem (2006) 281(21):14841–51. doi: 10.1074/jbc.M513737200 16551619

[29] Cuadrado A. Structural and Functional Characterization of Nrf2 Degradation by Glycogen Synthase Kinase 3/β-TrCP. Free Radical Biol Med (2015) 88:147–57. doi: 10.1016/j.freeradbiomed.2015.04.029 25937177

[30] Rojo AI Sagarra M Cuadrado A. GSK-3β Down-Regulates the Transcription Factor Nrf2 After Oxidant Damage: Relevance to Exposure of Neuronal Cells to Oxidative Stress. J Neurochem (2008) 105(1):192–202. doi: 10.1111/j.1471-4159.2007.05124.x 18005231

[31] Choi B-H Kim JM Kwak M-K. The Multifaceted Role of NRF2 in Cancer Progression and Cancer Stem Cells Maintenance. Arch Pharmacal Res (2021) 44(3):263–80. doi: 10.1007/s12272-021-01316-8 33754307

[32] Rojo de la Vega M Chapman E Zhang DD. NRF2 and the Hallmarks of Cancer. Cancer Cell (2018) 34(1):21–43. doi: 10.1016/j.ccell.2018.03.022 29731393 PMC6039250

[33] Torrente L DeNicola GM. Targeting NRF2 and Its Downstream Processes: Opportunities and Challenges. Annu Rev Pharmacol Toxicol (2022) 62:279–300. doi: 10.1146/annurev-pharmtox-052220-104025 34499527

[34] Jia Y Chen J Zhu H Jia ZH Cui MH. Aberrantly Elevated Redox Sensing Factor Nrf2 Promotes Cancer Stem Cell Survival *via* Enhanced Transcriptional Regulation of ABCG2 and Bcl-2/Bmi-1 Genes. Oncol Rep (2015) 34(5):2296–304. doi: 10.3892/or.2015.4214 26324021

[35] Kim D Choi BH Ryoo IG Kwak MK. High NRF2 Level Mediates Cancer Stem Cell-Like Properties of Aldehyde Dehydrogenase (ALDH)-High Ovarian Cancer Cells: Inhibitory Role of All-Trans Retinoic Acid in ALDH/NRF2 Signaling. Cell Death Dis (2018) 9(9):896. doi: 10.1038/s41419-018-0903-4 30166520 PMC6117306

[36] Ryoo IG Choi BH Ku SK Kwak MK. High CD44 Expression Mediates P62-Associated NFE2L2/NRF2 Activation in Breast Cancer Stem Cell-Like Cells: Implications for Cancer Stem Cell Resistance. Redox Biol (2018) 17:246–58. doi: 10.1016/j.redox.2018.04.015 PMC6006726 29729523

[37] Ryoo I-G Choi B-H Kwak M-K. Activation of NRF2 by P62 and Proteasome Reduction in Sphere-Forming Breast Carcinoma Cells. Oncotarget (2015) 6(10):8167–84. doi: 10.18632/oncotarget.3047 PMC4480743 25717032

[38] Zhu J Wang H Sun Q Ji X Zhu L Cong Z. Nrf2 is Required to Maintain the Self-Renewal of Glioma Stem Cells. BMC Cancer (2013) 13(1):380. doi: 10.1186/1471-2407-13-380 23937621 PMC3751732

[39] Wu T Harder BG Wong PK Lang JE Zhang DD. Oxidative Stress, Mammospheres and Nrf2-New Implication for Breast Cancer Therapy? Mol Carcinog (2015) 54(11):1494–502. doi: 10.1002/mc.22202 PMC4697962 25154499

[40] Ryu D Lee JH Kwak MK. NRF2 Level is Negatively Correlated With TGF-β1-Induced Lung Cancer Motility and Migration *via* NOX4-ROS Signaling. Arch Pharm Res (2020) 43(12):1297–310. doi: 10.1007/s12272-020-01298-z 33242180

[41] Choi JH Jin SW Lee GH Cho SM Jeong HG. Orostachys Japonicus Ethanol Extract Inhibits 2,4-Dinitrochlorobenzene-Induced Atopic Dermatitis-Like Skin Lesions in NC/Nga Mice and TNF-α/IFN-γ-Induced TARC Expression in HaCaT Cells. Toxicol Res (2020) 36(2):99–108. doi: 10.1007/s43188-019-00026-0 32257921 PMC7099123

[42] Hubert CG Rivera M Spangler LC Wu Q Mack SC Prager BC. A Three-Dimensional Organoid Culture System Derived From Human Glioblastomas Recapitulates the Hypoxic Gradients and Cancer Stem Cell Heterogeneity of Tumors Found *In Vivo* . Cancer Res (2016) 76(8):2465–77. doi: 10.1158/0008-5472.Can-15-2402 PMC4873351 26896279

[43] Shimozato O Waraya M Nakashima K Souda H Takiguchi N Yamamoto H. Receptor-Type Protein Tyrosine Phosphatase Kappa Directly Dephosphorylates CD133 and Regulates Downstream AKT Activation. Oncogene (2015) 34(15):1949–60. doi: 10.1038/onc.2014.141 24882578

[44] Wei Y Jiang Y Zou F Liu Y Wang S Xu N. Activation of PI3K/Akt Pathway by CD133-P85 Interaction Promotes Tumorigenic Capacity of Glioma Stem Cells. Proc Natl Acad Sci USA (2013) 110(17):6829–34. doi: 10.1073/pnas.1217002110 PMC3637720 23569237

[45] Hadnagy A Gaboury L Beaulieu R Balicki D. SP Analysis may be Used to Identify Cancer Stem Cell Populations. Exp Cell Res (2006) 312(19):3701–10. doi: 10.1016/j.yexcr.2006.08.030 17046749

[46] Bao S Wu Q McLendon RE Hao Y Shi Q Hjelmeland AB. Glioma Stem Cells Promote Radioresistance by Preferential Activation of the DNA Damage Response. Nature (2006) 444(7120):756. doi: 10.1038/nature05236 17051156

[47] Phillips TM McBride WH Pajonk F. The Response of CD24(-/Low)/CD44+ Breast Cancer-Initiating Cells to Radiation. J Natl Cancer Inst (2006) 98(24):1777–85. doi: 10.1093/jnci/djj495 17179479

[48] Herault O Hope KJ Deneault E Mayotte N Chagraoui J Wilhelm BT. A Role for GPx3 in Activity of Normal and Leukemia Stem Cells. J Exp Med (2012) 209(5):895–901. doi: 10.1084/jem.20102386 22508837 PMC3348115

[49] Naka K Hoshii T Muraguchi T Tadokoro Y Ooshio T Kondo Y. TGF-Beta-FOXO Signalling Maintains Leukaemia-Initiating Cells in Chronic Myeloid Leukaemia. Nature (2010) 463(7281):676–80. doi: 10.1038/nature08734 20130650

[50] Ishimoto T Nagano O Yae T Tamada M Motohara T Oshima H. CD44 Variant Regulates Redox Status in Cancer Cells by Stabilizing the xCT Subunit of System Xc(-) and Thereby Promotes Tumor Growth. Cancer Cell (2011) 19(3):387–400. doi: 10.1016/j.ccr.2011.01.038 21397861

[51] Dubrovska A Kim S Salamone RJ Walker JR Maira SM García-Echeverría C. The Role of PTEN/Akt/PI3K Signaling in the Maintenance and Viability of Prostate Cancer Stem-Like Cell Populations. Proc Natl Acad Sci USA (2009) 106(1):268–73. doi: 10.1073/pnas.0810956106 PMC2629188 19116269

[52] Chen J Shao R Li F Monteiro M Liu JP Xu ZP. PI3K/Akt/mTOR Pathway Dual Inhibitor BEZ235 Suppresses the Stemness of Colon Cancer Stem Cells. Clin Exp Pharmacol Physiol (2015) 42(12):1317–26. doi: 10.1111/1440-1681.12493 26399781

[53] Lien EC Lyssiotis CA Juvekar A Hu H Asara JM Cantley LC. Glutathione Biosynthesis is a Metabolic Vulnerability in PI(3)K/Akt-Driven Breast Cancer. Nat Cell Biol (2016) 18(5):572–8. doi: 10.1038/ncb3341 PMC4848114 27088857

[54] Mitsuishi Y Taguchi K Kawatani Y Shibata T Nukiwa T Aburatani H. Nrf2 Redirects Glucose and Glutamine Into Anabolic Pathways in Metabolic Reprogramming. Cancer Cell (2012) 22(1):66–79. doi: 10.1016/j.ccr.2012.05.016 22789539

[55] Kwak MK Itoh K Yamamoto M Kensler TW. Enhanced Expression of the Transcription Factor Nrf2 by Cancer Chemopreventive Agents: Role of Antioxidant Response Element-Like Sequences in the Nrf2 Promoter. Mol Cell Biol (2002) 22(9):2883–92. doi: 10.1128/mcb.22.9.2883-2892.2002 PMC133753 11940647

[56] Wu T Harder BG Wong PK Lang JE Zhang DD. Oxidative Stress, Mammospheres and Nrf2–new Implication for Breast Cancer Therapy? Mol Carcinog (2014) 54(11):1494–502. doi: 10.1002/mc.22202 PMC4697962 25154499

[57] Goto S Kawabata T Li TS. Enhanced Expression of ABCB1 and Nrf2 in CD133-Positive Cancer Stem Cells Associates With Doxorubicin Resistance. Stem Cells Int (2020) 2020:8868849. doi: 10.1155/2020/8868849 32849878 PMC7441449

