# Supplementary figures and images for: Role of CD133/NRF2 Axis in the Development of Colon Cancer Stem Cell-Like Properties
Source: Front Oncol. 2022 Jan 26;11:808300. doi: 10.3389/fonc.2021.808300 (PMC8825377; doi:10.3389/fonc.2021.808300)

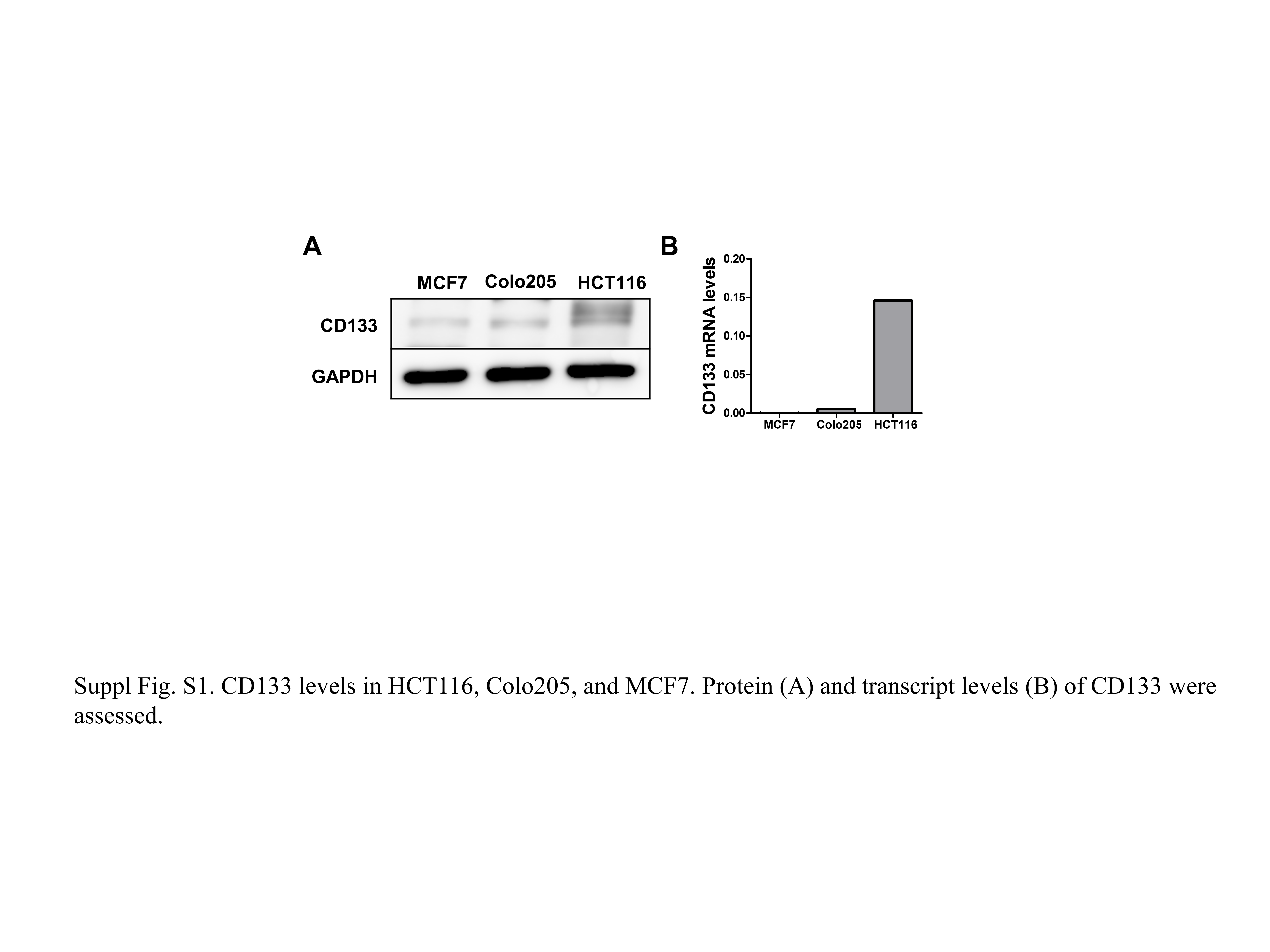

Supplement: Supplementary file 1 [file Image_1.tif]

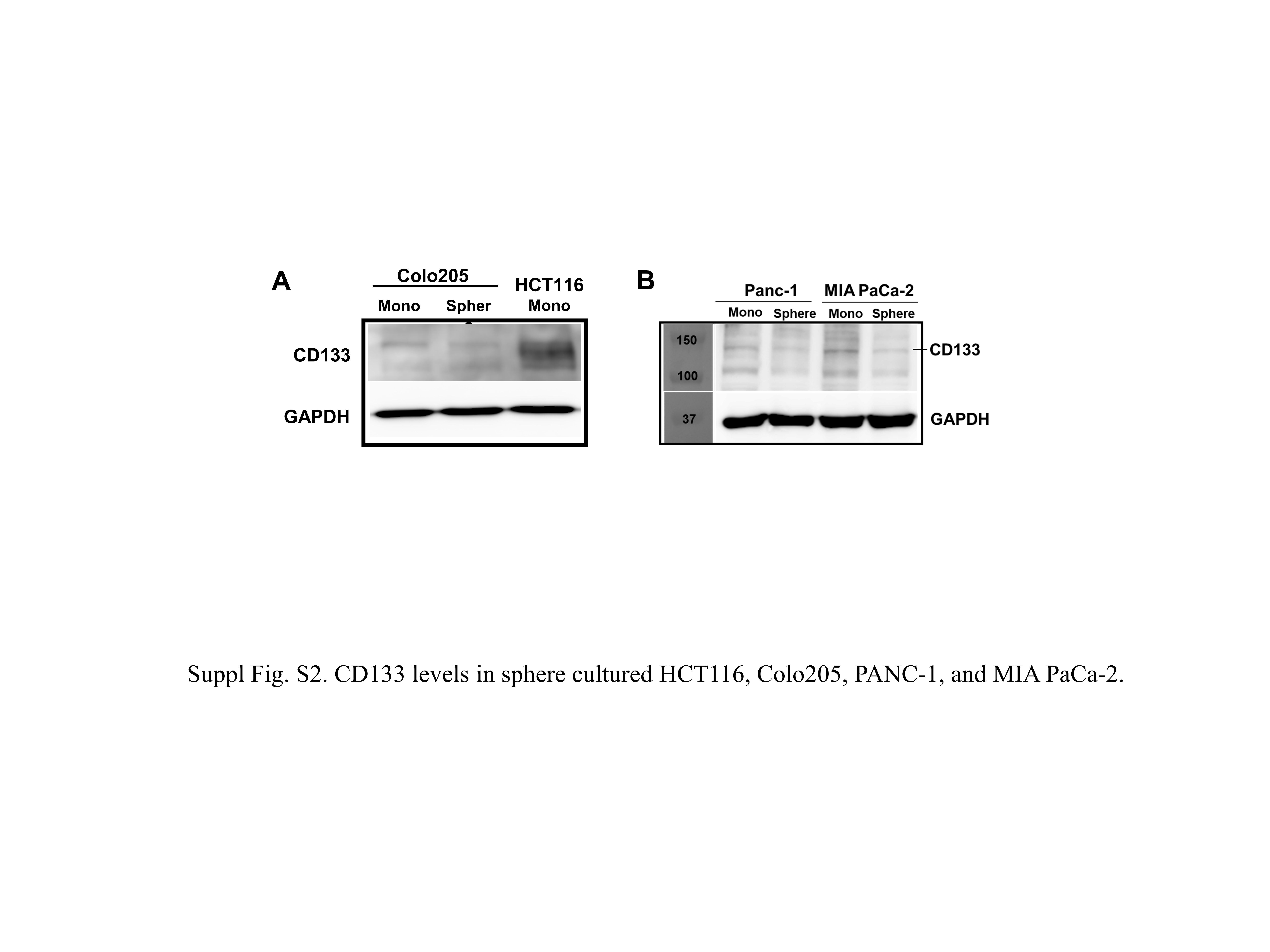

Supplement: Supplementary file 2 [file Image_2.tif]
